# 1-(3-Chloro­benz­yloxy)urea

**DOI:** 10.1107/S160053680904553X

**Published:** 2009-11-04

**Authors:** Xi Mai, Hong-Ying Xia, Yu-Sheng Cao, Wei Tong, Guo-Gang Tu

**Affiliations:** a State Key Laboratory of Food Science and Technology, Nanchang University, Nanchang 330047, People’s Republic of China; b Department of Pharmacy, Medical College of Nanchang University, Nanchang 330006, People’s Republic of China; c Department of Pharmacy, Shangrao Branch of Jiangxi Medical College, Shangrao 334000, People’s Republic of China; d Sino-German Joint Research Institute of Nanchang University, Nanchang 330006, People’s Republic of China

## Abstract

The asymmetric unit of the crystal structure of the title compound, C_8_H_9_ClN_2_O_2_, contains four independent mol­ecules. The dihedral angles between the urea N—(C=O)—N planes and the benzene rings are 83.3 (3), 87.8 (1), 89.1 (1) and 17.5 (2)° in the four mol­ecules. Extensive N—H⋯O hydrogen bonding is present in the crystal structure.

## Related literature

For general background to the design and synthesis of hydroxy­urea derivatives and their *in vitro* anti­tumor activity, see: Mai *et al.* (2009 ▶). For related structures, see: Armagan *et al.* (1976 ▶); Nielsen *et al.* (1993 ▶); Berman & Kim (1967 ▶); Howard *et al.* (1967 ▶); Larsen & Jerslev (1966 ▶); Thiessen *et al.* (1978 ▶); Yoshitaka *et al.* (1993 ▶).
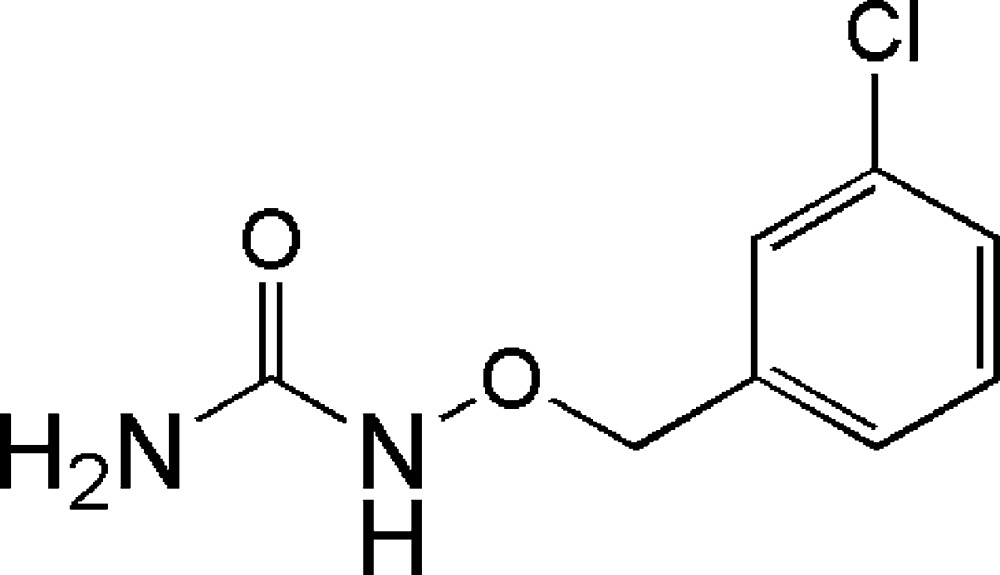


## Experimental

### 

#### Crystal data

C_8_H_9_ClN_2_O_2_
*M* *_r_* = 200.62
Triclinic, 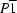

*a* = 10.830 (1) Å
*b* = 13.9410 (14) Å
*c* = 14.2750 (15) Å
α = 69.672 (1)°
β = 75.828 (2)°
γ = 70.388 (1)°
*V* = 1883.6 (3) Å^3^
*Z* = 8
Mo *K*α radiation
μ = 0.37 mm^−1^
*T* = 298 K
0.43 × 0.40 × 0.05 mm

#### Data collection

Bruker APEXII CCD area-detector diffractometer
Absorption correction: multi-scan (*SADABS*; Sheldrick, 1996 ▶) *T*_min_ = 0.856, *T*_max_ = 0.982
9908 measured reflections
6533 independent reflections
3124 reflections with *I* > 2σ(*I*)
*R*_int_ = 0.029

#### Refinement

*R*[*F* ^2^ > 2σ(*F* ^2^)] = 0.047
*wR*(*F* ^2^) = 0.094
*S* = 1.01
6533 reflections
469 parameters
H-atom parameters constrained
Δρ_max_ = 0.23 e Å^−3^
Δρ_min_ = −0.24 e Å^−3^

### 

Data collection: *APEX2* (Bruker, 2004 ▶); cell refinement: *SAINT* (Bruker, 2004 ▶); data reduction: *SAINT*; program(s) used to solve structure: *SHELXS97* (Sheldrick, 2008 ▶); program(s) used to refine structure: *SHELXL97* (Sheldrick, 2008 ▶); molecular graphics: *ORTEP-3 for Windows* (Farrugia, 1997 ▶); software used to prepare material for publication: *SHELXL97*.

## Supplementary Material

Crystal structure: contains datablocks I, global. DOI: 10.1107/S160053680904553X/xu2661sup1.cif

Structure factors: contains datablocks I. DOI: 10.1107/S160053680904553X/xu2661Isup2.hkl

Additional supplementary materials:  crystallographic information; 3D view; checkCIF report

## Figures and Tables

**Table 1:** Hydrogen-bond geometry (Å, °)

*D*—H⋯*A*	*D*—H	H⋯*A*	*D*⋯*A*	*D*—H⋯*A*
N1—H1⋯O5^i^	0.90	2.20	3.096 (3)	173
N2—H2*A*⋯O1^i^	0.86	2.16	3.023 (3)	177
N2—H2*B*⋯O3^ii^	0.86	2.29	2.971 (3)	136
N4—H4*A*⋯O7^iii^	0.86	2.11	2.971 (3)	176
N4—H4*B*⋯O5	0.86	2.39	3.017 (3)	130
N5—H5⋯O1^i^	0.90	2.19	3.090 (3)	176
N6—H6*A*⋯O5^iv^	0.86	2.07	2.925 (3)	177
N7—H7⋯O7^v^	0.90	2.04	2.937 (3)	171
N8—H8*A*⋯O3^ii^	0.86	2.09	2.947 (3)	177
N8—H8*B*⋯O1^i^	0.86	2.25	2.976 (3)	142

Symmetry codes: (i) 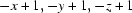; (ii) 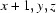; (iii) 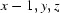; (iv) 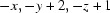; (v) 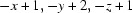.

**Fig. 1.**
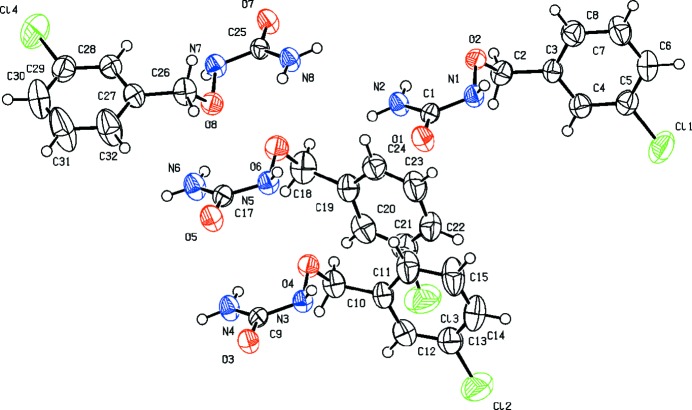
Molecular structure of the title compound showing the atom labelling scheme. Displacement ellipsoids are drawn at the 50% probability level.

**Fig. 2.**
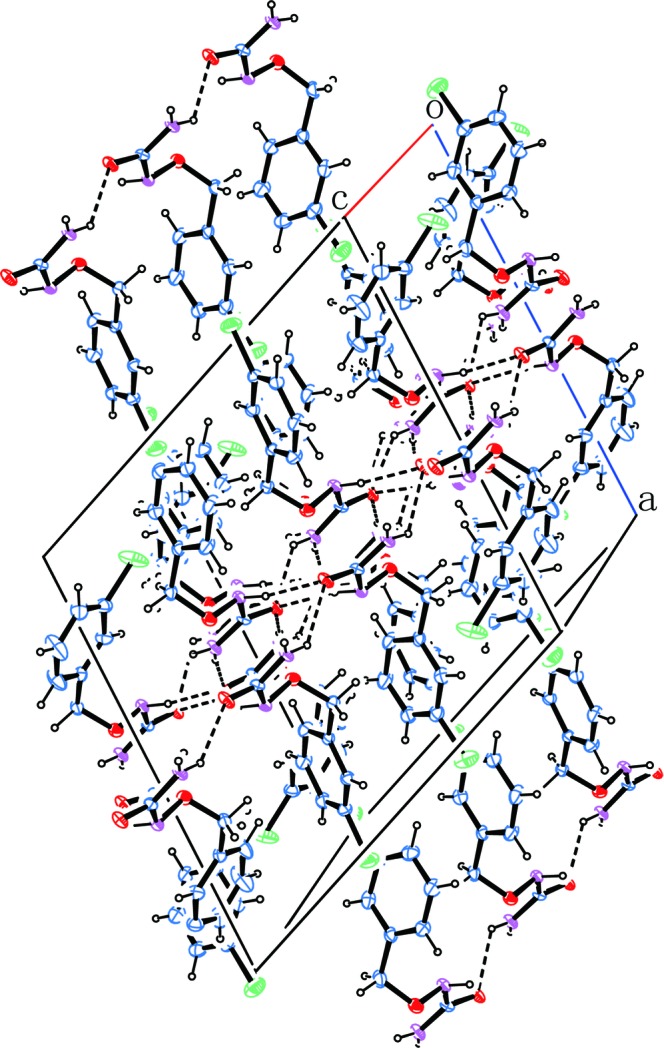
The unit cell diagram showing intermolecular hydrogen bonding as dashed lines

## supplementary crystallographic information

### Comment

Hydroxyurea (HU) is a substance used in cancer chemotherapy for many years, but it has several disadvantages, such as short half-life, extremely polar nature, the rapid development of resistance and so on. To obtain more potent compound, we have designed and synthesized HU derivatives, and evaluated their *in vitro* antitumor activities in our previous work (Mai *et al.*, 2009). Here we report the crystal structure of the title compound, 3-chlorobenzyloxyurea.

The structure of 3-chlorobenzyloxyurea is shown in Fig. 1. The conformations of the N–O and C=O bonds are opposite to each other, similar to that observed in N-hydroxyurea (Howard *et al.*, 1967; Thiessen *et al.*, 1978; Armagan *et al.*, 1976; Berman *et al.*, 1967; Larsen & Jerslev, 1966), 1-hydroxy-1-methylurea, 1-hydroxy-3-methylurea (Nielsen *et al.*, 1993), N-(6-phenoxy-2H-chromen-3-ylmethyl)-N-hydroxyurea (Yoshitaka *et al.*, 1993) and 1-(2-fluorobenzyl)-1-(2-fluorobenzyloxy) urea (Mai *et al.*, 2009). The bond parameters are similar to 1-(2-fluorobenzyl)-1-(2-fluorobenzyloxy)urea (Mai *et al.*, 2009). The asymmetric unit of the title compound contains four independent molecules. The dihedral angles between the urea N-(C=O)–N planes and benzene ring are 83.3 (3)°, 87.8 (1)°, 89.1 (1)° and 17.5 (2)° for the four molecules. The N–O bonds are twisted out of the urea N–(C=O)–N planes by 18.4 (3)°, 17.9 (3)°, 19.2 (4)° and -17.8 (3)°, respectively in the four molecules. In the crystal structure, molecules are linked through intermolecular N–H···O hydrogen bonds, forming the zigzig chain.

### Experimental

The title compound was synthesized by hydroxyurea (0.026 mol) with 3-chlorobenzyl chloride (0.034 mol) in methanol (80 ml) in the presence of potassium hydroxide (0.034 mol). After refluxing for 13 h, solvent was removed under reduced pressure at 308 K. The resulting crude solid was filtered and washed in trichloromethane, then recrystallized in acetone and trichloromethane solution (5:2), filtered and dried. Colorless platelet single crystals of the title compound were recrystallized from the mixed solvent acetone and n-hexane (5:10).

### Refinement

H atoms were placed in calculated positions with N—H = 0.90 (imino), 0.86 Å (amino), C—H = 0.93 (aromatic) and 0.97 Å (methylene), and refined in riding mode with U_iso_(H) = 1.2U_eq_(C,N).

### Crystal data

**Table:**

C_8_H_9_ClN_2_O_2_	*Z* = 8
*M**_r_* = 200.62	*F*(000) = 832
Triclinic, *P*1	*D*_x_ = 1.415 Mg m^−^^3^
Hall symbol: -P 1	Mo *K*α radiation, λ = 0.71073 Å
*a* = 10.830 (1) Å	Cell parameters from 1978 reflections
*b* = 13.9410 (14) Å	θ = 2.2–22.6°
*c* = 14.2750 (15) Å	µ = 0.37 mm^−^^1^
α = 69.672 (1)°	*T* = 298 K
β = 75.828 (2)°	Platelet, colourless
γ = 70.388 (1)°	0.43 × 0.40 × 0.05 mm
*V* = 1883.6 (3) Å^3^	

### Data collection

**Table:**

Bruker APEXII CCD area-detector diffractometer	6533 independent reflections
Radiation source: fine-focus sealed tube	3124 reflections with *I* > 2σ(*I*)
graphite	*R*_int_ = 0.029
φ and ω scans	θ_max_ = 25.0°, θ_min_ = 1.5°
Absorption correction: multi-scan (*SADABS*; Sheldrick, 1996)	*h* = −12→11
*T*_min_ = 0.856, *T*_max_ = 0.982	*k* = −16→16
9908 measured reflections	*l* = −16→16

### Refinement

**Table:**

Refinement on *F*^2^	Primary atom site location: structure-invariant direct methods
Least-squares matrix: full	Secondary atom site location: difference Fourier map
*R*[*F*^2^ > 2σ(*F*^2^)] = 0.047	Hydrogen site location: inferred from neighbouring sites
*wR*(*F*^2^) = 0.094	H-atom parameters constrained
*S* = 1.01	*w* = 1/[σ^2^(*F*_o_^2^) + (0.0205*P*)^2^] where *P* = (*F*_o_^2^ + 2*F*_c_^2^)/3
6533 reflections	(Δ/σ)_max_ = 0.001
469 parameters	Δρ_max_ = 0.23 e Å^−^^3^
0 restraints	Δρ_min_ = −0.24 e Å^−^^3^

### Special details

**Table:**

Geometry. All e.s.d.'s (except the e.s.d. in the dihedral angle between two l.s. planes) are estimated using the full covariance matrix. The cell e.s.d.'s are taken into account individually in the estimation of e.s.d.'s in distances, angles and torsion angles; correlations between e.s.d.'s in cell parameters are only used when they are defined by crystal symmetry. An approximate (isotropic) treatment of cell e.s.d.'s is used for estimating e.s.d.'s involving l.s. planes.
Refinement. Refinement of *F*^2^ against ALL reflections. The weighted *R*-factor *wR* and goodness of fit *S* are based on *F*^2^, conventional *R*-factors *R* are based on *F*, with *F* set to zero for negative *F*^2^. The threshold expression of *F*^2^ > σ(*F*^2^) is used only for calculating *R*-factors(gt) *etc*. and is not relevant to the choice of reflections for refinement. *R*-factors based on *F*^2^ are statistically about twice as large as those based on *F*, and *R*- factors based on ALL data will be even larger.

### Fractional atomic coordinates and isotropic or equivalent isotropic displacement parameters (Å^2^)

**Table:**

	*x*	*y*	*z*	*U*_iso_*/*U*_eq_	
Cl1	1.11832 (10)	0.31531 (8)	−0.02808 (6)	0.0839 (3)	
Cl2	0.25832 (11)	0.58558 (9)	−0.04798 (7)	0.0998 (4)	
Cl3	0.39222 (11)	0.83726 (9)	−0.04386 (7)	0.1020 (4)	
Cl4	0.16163 (12)	1.13164 (10)	0.84040 (10)	0.1271 (5)	
N1	0.8146 (2)	0.36994 (18)	0.39732 (16)	0.0401 (6)	
H1	0.8418	0.3029	0.4370	0.048*	
N2	0.6676 (2)	0.51825 (17)	0.44071 (16)	0.0419 (6)	
H2A	0.5900	0.5522	0.4631	0.050*	
H2B	0.7301	0.5491	0.4177	0.050*	
N3	0.0506 (2)	0.61462 (18)	0.39981 (16)	0.0380 (6)	
H3	0.0757	0.5489	0.4424	0.046*	
N4	−0.0889 (2)	0.76897 (17)	0.43587 (16)	0.0453 (7)	
H4A	−0.1647	0.8054	0.4583	0.054*	
H4B	−0.0251	0.7980	0.4089	0.054*	
N5	0.2948 (2)	0.86095 (18)	0.39097 (17)	0.0438 (6)	
H5	0.3203	0.7948	0.4327	0.053*	
N6	0.1619 (2)	1.01583 (18)	0.42966 (17)	0.0529 (7)	
H6A	0.0882	1.0524	0.4549	0.063*	
H6B	0.2252	1.0448	0.3991	0.063*	
N7	0.4342 (2)	0.89042 (18)	0.59283 (16)	0.0422 (6)	
H7	0.4119	0.9574	0.5520	0.051*	
N8	0.5745 (2)	0.73892 (17)	0.55203 (16)	0.0447 (6)	
H8A	0.6513	0.7025	0.5309	0.054*	
H8B	0.5093	0.7111	0.5735	0.054*	
O1	0.60499 (19)	0.36862 (15)	0.47386 (15)	0.0501 (6)	
O2	0.91190 (18)	0.42284 (15)	0.38500 (13)	0.0421 (5)	
O3	−0.15777 (19)	0.62023 (14)	0.48081 (14)	0.0462 (5)	
O4	0.15349 (18)	0.66423 (15)	0.38002 (14)	0.0440 (5)	
O5	0.09142 (19)	0.86693 (15)	0.48084 (15)	0.0524 (6)	
O6	0.4015 (2)	0.90763 (15)	0.36655 (15)	0.0510 (6)	
O7	0.64654 (19)	0.88364 (14)	0.52150 (14)	0.0469 (5)	
O8	0.33178 (18)	0.84381 (15)	0.60348 (14)	0.0458 (5)	
C1	0.6909 (3)	0.4182 (2)	0.4417 (2)	0.0379 (7)	
C2	0.9543 (3)	0.4670 (2)	0.2794 (2)	0.0463 (8)	
H2C	0.9940	0.5227	0.2714	0.056*	
H2D	0.8774	0.4992	0.2444	0.056*	
C3	1.0527 (3)	0.3856 (2)	0.2309 (2)	0.0400 (8)	
C4	1.0418 (3)	0.3863 (2)	0.1359 (2)	0.0473 (8)	
H4	0.9715	0.4348	0.1032	0.057*	
C5	1.1353 (3)	0.3151 (3)	0.0903 (2)	0.0489 (8)	
C6	1.2408 (3)	0.2433 (3)	0.1353 (2)	0.0573 (9)	
H6	1.3038	0.1964	0.1028	0.069*	
C7	1.2522 (3)	0.2418 (3)	0.2306 (3)	0.0619 (10)	
H7A	1.3230	0.1931	0.2627	0.074*	
C8	1.1592 (3)	0.3119 (3)	0.2776 (2)	0.0551 (9)	
H8	1.1675	0.3100	0.3416	0.066*	
C9	−0.0704 (3)	0.6681 (2)	0.4428 (2)	0.0373 (7)	
C10	0.1852 (3)	0.7051 (2)	0.2732 (2)	0.0506 (9)	
H10A	0.2443	0.7490	0.2591	0.061*	
H10B	0.1045	0.7503	0.2456	0.061*	
C11	0.2494 (3)	0.6202 (3)	0.2204 (2)	0.0462 (8)	
C12	0.2222 (3)	0.6366 (3)	0.1247 (2)	0.0552 (9)	
H12	0.1590	0.6975	0.0957	0.066*	
C13	0.2892 (4)	0.5626 (3)	0.0729 (2)	0.0578 (9)	
C14	0.3784 (4)	0.4728 (3)	0.1150 (3)	0.0694 (11)	
H14	0.4233	0.4234	0.0795	0.083*	
C15	0.4031 (4)	0.4544 (3)	0.2107 (3)	0.0804 (12)	
H15	0.4632	0.3915	0.2403	0.096*	
C16	0.3399 (3)	0.5278 (3)	0.2632 (2)	0.0659 (10)	
H16	0.3582	0.5151	0.3275	0.079*	
C17	0.1778 (3)	0.9153 (3)	0.4379 (2)	0.0424 (8)	
C18	0.4308 (3)	0.9459 (3)	0.2589 (2)	0.0649 (10)	
H18A	0.5039	0.9773	0.2420	0.078*	
H18B	0.3544	1.0016	0.2333	0.078*	
C19	0.4666 (3)	0.8607 (3)	0.2068 (2)	0.0498 (9)	
C20	0.4215 (3)	0.8854 (3)	0.1150 (3)	0.0608 (10)	
H20	0.3710	0.9537	0.0858	0.073*	
C21	0.4533 (3)	0.8067 (3)	0.0688 (2)	0.0625 (10)	
C22	0.5279 (3)	0.7067 (3)	0.1085 (3)	0.0665 (10)	
H22	0.5489	0.6552	0.0754	0.080*	
C23	0.5723 (3)	0.6827 (3)	0.1992 (3)	0.0687 (10)	
H23	0.6225	0.6141	0.2278	0.082*	
C24	0.5427 (3)	0.7592 (3)	0.2471 (2)	0.0575 (9)	
H24	0.5744	0.7424	0.3074	0.069*	
C25	0.5569 (3)	0.8378 (2)	0.5515 (2)	0.0364 (7)	
C26	0.2837 (3)	0.8033 (2)	0.7081 (2)	0.0504 (9)	
H26A	0.3582	0.7727	0.7456	0.061*	
H26B	0.2461	0.7465	0.7157	0.061*	
C27	0.1814 (3)	0.8842 (2)	0.7541 (2)	0.0443 (8)	
C28	0.2123 (3)	0.9650 (3)	0.7696 (2)	0.0560 (9)	
H28	0.2968	0.9742	0.7459	0.067*	
C29	0.1197 (4)	1.0319 (3)	0.8198 (3)	0.0605 (10)	
C30	−0.0046 (4)	1.0205 (3)	0.8561 (3)	0.0815 (12)	
H30	−0.0658	1.0643	0.8926	0.098*	
C31	−0.0373 (4)	0.9426 (3)	0.8373 (4)	0.1146 (18)	
H31	−0.1230	0.9356	0.8587	0.137*	
C32	0.0543 (4)	0.8753 (3)	0.7876 (3)	0.0877 (13)	
H32	0.0304	0.8226	0.7763	0.105*	

### Atomic displacement parameters (Å^2^)

**Table:**

	*U*^11^	*U*^22^	*U*^33^	*U*^12^	*U*^13^	*U*^23^
Cl1	0.1008 (8)	0.0997 (8)	0.0604 (6)	−0.0235 (7)	−0.0021 (6)	−0.0446 (6)
Cl2	0.0901 (8)	0.1568 (11)	0.0720 (7)	−0.0397 (8)	−0.0086 (6)	−0.0533 (7)
Cl3	0.1091 (9)	0.1371 (11)	0.0636 (6)	−0.0405 (8)	−0.0252 (6)	−0.0189 (6)
Cl4	0.1133 (10)	0.1276 (11)	0.1915 (13)	−0.0182 (8)	−0.0241 (10)	−0.1208 (10)
N1	0.0363 (16)	0.0381 (16)	0.0505 (15)	−0.0135 (14)	0.0008 (13)	−0.0199 (13)
N2	0.0341 (15)	0.0312 (16)	0.0625 (16)	−0.0105 (13)	0.0005 (13)	−0.0198 (13)
N3	0.0380 (16)	0.0337 (15)	0.0433 (15)	−0.0127 (13)	−0.0003 (13)	−0.0134 (12)
N4	0.0397 (16)	0.0333 (17)	0.0641 (17)	−0.0126 (13)	0.0039 (13)	−0.0209 (13)
N5	0.0432 (17)	0.0370 (17)	0.0543 (16)	−0.0154 (14)	−0.0014 (14)	−0.0168 (13)
N6	0.0454 (17)	0.0355 (17)	0.0766 (19)	−0.0135 (14)	0.0034 (15)	−0.0211 (14)
N7	0.0389 (16)	0.0341 (16)	0.0532 (16)	−0.0117 (14)	−0.0013 (14)	−0.0144 (13)
N8	0.0382 (16)	0.0316 (16)	0.0665 (17)	−0.0097 (13)	−0.0005 (13)	−0.0218 (13)
O1	0.0376 (13)	0.0378 (13)	0.0820 (16)	−0.0174 (11)	0.0014 (12)	−0.0260 (11)
O2	0.0374 (12)	0.0513 (14)	0.0452 (12)	−0.0174 (11)	0.0006 (10)	−0.0226 (10)
O3	0.0372 (13)	0.0399 (13)	0.0690 (14)	−0.0186 (11)	0.0037 (11)	−0.0250 (11)
O4	0.0387 (13)	0.0486 (14)	0.0505 (13)	−0.0196 (11)	0.0025 (11)	−0.0201 (11)
O5	0.0414 (14)	0.0408 (14)	0.0827 (16)	−0.0187 (12)	0.0033 (12)	−0.0283 (12)
O6	0.0466 (14)	0.0544 (15)	0.0578 (14)	−0.0230 (12)	0.0024 (11)	−0.0215 (11)
O7	0.0359 (13)	0.0333 (13)	0.0743 (14)	−0.0120 (11)	−0.0013 (11)	−0.0211 (11)
O8	0.0391 (13)	0.0521 (14)	0.0522 (13)	−0.0186 (11)	0.0001 (11)	−0.0212 (11)
C1	0.040 (2)	0.034 (2)	0.0448 (19)	−0.0074 (17)	−0.0077 (17)	−0.0187 (16)
C2	0.051 (2)	0.046 (2)	0.0417 (19)	−0.0195 (18)	0.0019 (17)	−0.0133 (16)
C3	0.042 (2)	0.039 (2)	0.0374 (18)	−0.0149 (17)	0.0032 (16)	−0.0115 (15)
C4	0.047 (2)	0.043 (2)	0.050 (2)	−0.0110 (17)	−0.0046 (17)	−0.0145 (17)
C5	0.053 (2)	0.051 (2)	0.045 (2)	−0.0189 (19)	0.0003 (18)	−0.0179 (17)
C6	0.061 (3)	0.047 (2)	0.057 (2)	−0.012 (2)	0.011 (2)	−0.0221 (18)
C7	0.056 (2)	0.055 (2)	0.058 (2)	0.0005 (19)	−0.004 (2)	−0.0146 (19)
C8	0.054 (2)	0.060 (3)	0.047 (2)	−0.012 (2)	−0.0039 (19)	−0.0160 (18)
C9	0.037 (2)	0.037 (2)	0.0447 (19)	−0.0107 (17)	−0.0061 (16)	−0.0193 (16)
C10	0.051 (2)	0.042 (2)	0.050 (2)	−0.0157 (17)	0.0065 (18)	−0.0099 (17)
C11	0.045 (2)	0.044 (2)	0.043 (2)	−0.0166 (18)	0.0088 (17)	−0.0103 (17)
C12	0.046 (2)	0.053 (2)	0.059 (2)	−0.0114 (18)	0.0005 (19)	−0.0160 (19)
C13	0.055 (2)	0.064 (3)	0.050 (2)	−0.022 (2)	0.0023 (19)	−0.014 (2)
C14	0.086 (3)	0.058 (3)	0.056 (2)	−0.022 (2)	0.021 (2)	−0.025 (2)
C15	0.096 (3)	0.047 (3)	0.056 (3)	0.014 (2)	0.005 (2)	−0.008 (2)
C16	0.076 (3)	0.053 (3)	0.045 (2)	0.004 (2)	−0.002 (2)	−0.0105 (19)
C17	0.041 (2)	0.038 (2)	0.053 (2)	−0.0084 (18)	−0.0080 (18)	−0.0211 (17)
C18	0.075 (3)	0.052 (2)	0.062 (2)	−0.027 (2)	0.011 (2)	−0.013 (2)
C19	0.051 (2)	0.046 (2)	0.047 (2)	−0.0181 (19)	0.0096 (18)	−0.0128 (18)
C20	0.054 (2)	0.053 (2)	0.057 (2)	−0.0084 (19)	0.001 (2)	−0.006 (2)
C21	0.053 (2)	0.080 (3)	0.048 (2)	−0.021 (2)	0.0020 (19)	−0.015 (2)
C22	0.062 (3)	0.076 (3)	0.060 (2)	−0.014 (2)	0.006 (2)	−0.034 (2)
C23	0.066 (3)	0.060 (3)	0.063 (2)	0.004 (2)	−0.005 (2)	−0.020 (2)
C24	0.057 (2)	0.057 (3)	0.052 (2)	−0.009 (2)	−0.0074 (19)	−0.015 (2)
C25	0.036 (2)	0.030 (2)	0.0425 (18)	−0.0054 (17)	−0.0067 (16)	−0.0129 (15)
C26	0.054 (2)	0.041 (2)	0.054 (2)	−0.0180 (18)	0.0034 (18)	−0.0124 (17)
C27	0.039 (2)	0.043 (2)	0.0460 (19)	−0.0100 (17)	−0.0013 (16)	−0.0103 (16)
C28	0.043 (2)	0.069 (3)	0.063 (2)	−0.015 (2)	−0.0005 (18)	−0.033 (2)
C29	0.067 (3)	0.056 (3)	0.060 (2)	−0.009 (2)	−0.011 (2)	−0.0252 (19)
C30	0.078 (3)	0.057 (3)	0.083 (3)	−0.002 (2)	0.022 (2)	−0.026 (2)
C31	0.061 (3)	0.078 (3)	0.195 (5)	−0.027 (3)	0.048 (3)	−0.067 (3)
C32	0.060 (3)	0.062 (3)	0.142 (4)	−0.025 (2)	0.024 (3)	−0.049 (3)

### Geometric parameters (Å, °)

**Table:**

Cl1—C5	1.742 (3)	C6—C7	1.388 (4)
Cl2—C13	1.737 (3)	C6—H6	0.9300
Cl3—C21	1.750 (3)	C7—C8	1.372 (4)
Cl4—C29	1.732 (3)	C7—H7A	0.9300
N1—C1	1.387 (3)	C8—H8	0.9300
N1—O2	1.424 (2)	C10—C11	1.504 (4)
N1—H1	0.9000	C10—H10A	0.9700
N2—C1	1.327 (3)	C10—H10B	0.9700
N2—H2A	0.8600	C11—C16	1.376 (4)
N2—H2B	0.8600	C11—C12	1.392 (4)
N3—C9	1.385 (3)	C12—C13	1.380 (4)
N3—O4	1.424 (2)	C12—H12	0.9300
N3—H3	0.9000	C13—C14	1.346 (4)
N4—C9	1.323 (3)	C14—C15	1.376 (4)
N4—H4A	0.8600	C14—H14	0.9300
N4—H4B	0.8600	C15—C16	1.375 (4)
N5—C17	1.386 (3)	C15—H15	0.9300
N5—O6	1.426 (3)	C16—H16	0.9300
N5—H5	0.9000	C18—C19	1.510 (4)
N6—C17	1.320 (3)	C18—H18A	0.9700
N6—H6A	0.8600	C18—H18B	0.9700
N6—H6B	0.8600	C19—C24	1.377 (4)
N7—C25	1.384 (3)	C19—C20	1.400 (4)
N7—O8	1.417 (2)	C20—C21	1.378 (4)
N7—H7	0.9000	C20—H20	0.9300
N8—C25	1.324 (3)	C21—C22	1.355 (4)
N8—H8A	0.8600	C22—C23	1.383 (4)
N8—H8B	0.8600	C22—H22	0.9300
O1—C1	1.247 (3)	C23—C24	1.371 (4)
O2—C2	1.439 (3)	C23—H23	0.9300
O3—C9	1.247 (3)	C24—H24	0.9300
O4—C10	1.428 (3)	C26—C27	1.500 (4)
O5—C17	1.249 (3)	C26—H26A	0.9700
O6—C18	1.432 (3)	C26—H26B	0.9700
O7—C25	1.248 (3)	C27—C32	1.374 (4)
O8—C26	1.432 (3)	C27—C28	1.377 (4)
C2—C3	1.507 (4)	C28—C29	1.369 (4)
C2—H2C	0.9700	C28—H28	0.9300
C2—H2D	0.9700	C29—C30	1.360 (4)
C3—C4	1.387 (4)	C30—C31	1.372 (5)
C3—C8	1.391 (4)	C30—H30	0.9300
C4—C5	1.374 (4)	C31—C32	1.364 (5)
C4—H4	0.9300	C31—H31	0.9300
C5—C6	1.366 (4)	C32—H32	0.9300
			
C1—N1—O2	113.2 (2)	C13—C12—H12	120.0
C1—N1—H1	108.3	C11—C12—H12	120.0
O2—N1—H1	108.2	C14—C13—C12	120.6 (3)
C1—N2—H2A	120.0	C14—C13—Cl2	119.7 (3)
C1—N2—H2B	120.0	C12—C13—Cl2	119.7 (3)
H2A—N2—H2B	120.0	C13—C14—C15	119.8 (3)
C9—N3—O4	114.3 (2)	C13—C14—H14	120.1
C9—N3—H3	108.1	C15—C14—H14	120.1
O4—N3—H3	108.1	C16—C15—C14	120.8 (3)
C9—N4—H4A	120.0	C16—C15—H15	119.6
C9—N4—H4B	120.0	C14—C15—H15	119.6
H4A—N4—H4B	120.0	C15—C16—C11	119.8 (3)
C17—N5—O6	114.9 (2)	C15—C16—H16	120.1
C17—N5—H5	108.0	C11—C16—H16	120.1
O6—N5—H5	107.9	O5—C17—N6	124.3 (3)
C17—N6—H6A	120.0	O5—C17—N5	117.3 (3)
C17—N6—H6B	120.0	N6—C17—N5	118.2 (3)
H6A—N6—H6B	120.0	O6—C18—C19	113.6 (2)
C25—N7—O8	114.0 (2)	O6—C18—H18A	108.8
C25—N7—H7	107.9	C19—C18—H18A	108.8
O8—N7—H7	107.9	O6—C18—H18B	108.8
C25—N8—H8A	120.0	C19—C18—H18B	108.8
C25—N8—H8B	120.0	H18A—C18—H18B	107.7
H8A—N8—H8B	120.0	C24—C19—C20	119.1 (3)
N1—O2—C2	110.04 (18)	C24—C19—C18	121.7 (3)
N3—O4—C10	108.3 (2)	C20—C19—C18	119.2 (3)
N5—O6—C18	108.6 (2)	C21—C20—C19	118.7 (3)
N7—O8—C26	110.3 (2)	C21—C20—H20	120.7
O1—C1—N2	123.4 (3)	C19—C20—H20	120.7
O1—C1—N1	118.8 (3)	C22—C21—C20	122.2 (3)
N2—C1—N1	117.6 (3)	C22—C21—Cl3	119.4 (3)
O2—C2—C3	113.2 (2)	C20—C21—Cl3	118.4 (3)
O2—C2—H2C	108.9	C21—C22—C23	118.9 (3)
C3—C2—H2C	108.9	C21—C22—H22	120.6
O2—C2—H2D	108.9	C23—C22—H22	120.6
C3—C2—H2D	108.9	C24—C23—C22	120.5 (3)
H2C—C2—H2D	107.7	C24—C23—H23	119.8
C4—C3—C8	118.5 (3)	C22—C23—H23	119.8
C4—C3—C2	120.1 (3)	C23—C24—C19	120.6 (3)
C8—C3—C2	121.4 (3)	C23—C24—H24	119.7
C5—C4—C3	119.8 (3)	C19—C24—H24	119.7
C5—C4—H4	120.1	O7—C25—N8	123.8 (3)
C3—C4—H4	120.1	O7—C25—N7	118.3 (3)
C6—C5—C4	121.9 (3)	N8—C25—N7	117.8 (3)
C6—C5—Cl1	118.9 (3)	O8—C26—C27	114.8 (2)
C4—C5—Cl1	119.2 (3)	O8—C26—H26A	108.6
C5—C6—C7	118.7 (3)	C27—C26—H26A	108.6
C5—C6—H6	120.7	O8—C26—H26B	108.6
C7—C6—H6	120.7	C27—C26—H26B	108.6
C8—C7—C6	120.2 (3)	H26A—C26—H26B	107.5
C8—C7—H7A	119.9	C32—C27—C28	117.9 (3)
C6—C7—H7A	119.9	C32—C27—C26	120.1 (3)
C7—C8—C3	121.0 (3)	C28—C27—C26	121.9 (3)
C7—C8—H8	119.5	C29—C28—C27	120.5 (3)
C3—C8—H8	119.5	C29—C28—H28	119.8
O3—C9—N4	123.9 (3)	C27—C28—H28	119.8
O3—C9—N3	118.1 (3)	C30—C29—C28	121.4 (3)
N4—C9—N3	117.9 (3)	C30—C29—Cl4	118.9 (3)
O4—C10—C11	113.7 (2)	C28—C29—Cl4	119.7 (3)
O4—C10—H10A	108.8	C29—C30—C31	118.2 (4)
C11—C10—H10A	108.8	C29—C30—H30	120.9
O4—C10—H10B	108.8	C31—C30—H30	120.9
C11—C10—H10B	108.8	C32—C31—C30	120.8 (4)
H10A—C10—H10B	107.7	C32—C31—H31	119.6
C16—C11—C12	119.0 (3)	C30—C31—H31	119.6
C16—C11—C10	120.9 (3)	C31—C32—C27	121.1 (4)
C12—C11—C10	120.0 (3)	C31—C32—H32	119.5
C13—C12—C11	120.0 (3)	C27—C32—H32	119.5
			
C1—N1—O2—C2	−114.4 (2)	C12—C11—C16—C15	0.9 (5)
C9—N3—O4—C10	−110.6 (3)	C10—C11—C16—C15	−176.0 (3)
C17—N5—O6—C18	−112.7 (3)	O6—N5—C17—O5	−164.4 (2)
C25—N7—O8—C26	114.6 (3)	O6—N5—C17—N6	19.2 (4)
O2—N1—C1—O1	−166.4 (2)	N5—O6—C18—C19	−58.1 (3)
O2—N1—C1—N2	18.4 (3)	O6—C18—C19—C24	−38.7 (4)
N1—O2—C2—C3	−79.1 (3)	O6—C18—C19—C20	141.4 (3)
O2—C2—C3—C4	137.8 (3)	C24—C19—C20—C21	0.9 (5)
O2—C2—C3—C8	−45.1 (4)	C18—C19—C20—C21	−179.2 (3)
C8—C3—C4—C5	−0.1 (4)	C19—C20—C21—C22	−0.8 (5)
C2—C3—C4—C5	177.0 (3)	C19—C20—C21—Cl3	178.1 (2)
C3—C4—C5—C6	−0.7 (5)	C20—C21—C22—C23	0.8 (5)
C3—C4—C5—Cl1	178.8 (2)	Cl3—C21—C22—C23	−178.0 (3)
C4—C5—C6—C7	0.9 (5)	C21—C22—C23—C24	−0.9 (5)
Cl1—C5—C6—C7	−178.5 (2)	C22—C23—C24—C19	1.1 (5)
C5—C6—C7—C8	−0.5 (5)	C20—C19—C24—C23	−1.1 (5)
C6—C7—C8—C3	−0.3 (5)	C18—C19—C24—C23	179.0 (3)
C4—C3—C8—C7	0.5 (5)	O8—N7—C25—O7	166.5 (2)
C2—C3—C8—C7	−176.5 (3)	O8—N7—C25—N8	−17.8 (3)
O4—N3—C9—O3	−165.9 (2)	N7—O8—C26—C27	83.7 (3)
O4—N3—C9—N4	17.9 (3)	O8—C26—C27—C32	114.8 (3)
N3—O4—C10—C11	−67.9 (3)	O8—C26—C27—C28	−69.1 (4)
O4—C10—C11—C16	−38.4 (4)	C32—C27—C28—C29	2.0 (5)
O4—C10—C11—C12	144.7 (3)	C26—C27—C28—C29	−174.2 (3)
C16—C11—C12—C13	−2.2 (5)	C27—C28—C29—C30	0.3 (5)
C10—C11—C12—C13	174.7 (3)	C27—C28—C29—Cl4	178.9 (2)
C11—C12—C13—C14	1.7 (5)	C28—C29—C30—C31	−2.8 (6)
C11—C12—C13—Cl2	−178.1 (2)	Cl4—C29—C30—C31	178.6 (3)
C12—C13—C14—C15	0.2 (5)	C29—C30—C31—C32	2.9 (7)
Cl2—C13—C14—C15	180.0 (3)	C30—C31—C32—C27	−0.7 (7)
C13—C14—C15—C16	−1.6 (6)	C28—C27—C32—C31	−1.8 (6)
C14—C15—C16—C11	1.0 (6)	C26—C27—C32—C31	174.4 (4)

### Hydrogen-bond geometry (Å, °)

**Table:**

*D*—H···*A*	*D*—H	H···*A*	*D*···*A*	*D*—H···*A*
N1—H1···O5^i^	0.90	2.20	3.096 (3)	173
N2—H2A···O1^i^	0.86	2.16	3.023 (3)	177
N2—H2B···O3^ii^	0.86	2.29	2.971 (3)	136
N4—H4A···O7^iii^	0.86	2.11	2.971 (3)	176
N4—H4B···O5	0.86	2.39	3.017 (3)	130
N5—H5···O1^i^	0.90	2.19	3.090 (3)	176
N6—H6A···O5^iv^	0.86	2.07	2.925 (3)	177
N7—H7···O7^v^	0.90	2.04	2.937 (3)	171
N8—H8A···O3^ii^	0.86	2.09	2.947 (3)	177
N8—H8B···O1^i^	0.86	2.25	2.976 (3)	142

Symmetry codes: (i) −*x*+1, −*y*+1, −*z*+1; (ii) *x*+1, *y*, *z*; (iii) *x*−1, *y*, *z*; (iv) −*x*, −*y*+2, −*z*+1; (v) −*x*+1, −*y*+2, −*z*+1.
